# Obituary

**DOI:** 10.1155/S1463924693000070

**Published:** 1993


Journal of Automatic Chemistry, Vol. 15, No. 2 (March–April 1993), pp. 35–36

### Obituary

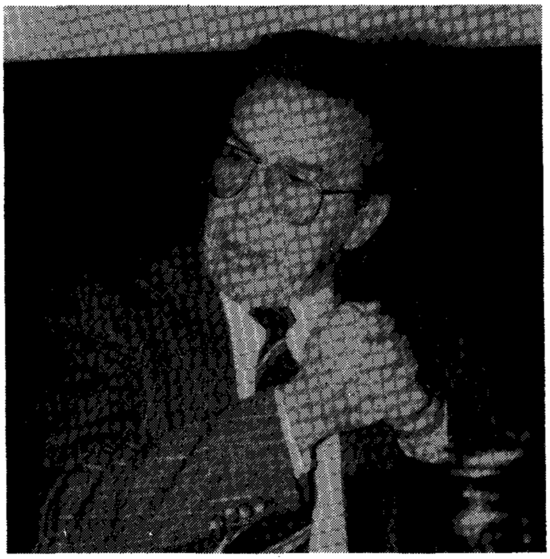

**Wilhelm Simon**
**1929–1992**

Sadly, Wilhelm Simon passed away on 17 November 1992. He was an analytical chemist with a worldwide reputation. His name was principally connected with research in the area of chemical sensors. However, the list of his over 450 scientific publications and well over 500 speeches clearly demonstrates that he made relevant contributions to many different areas of analytical chemistry. Aside from the wide range of his interests, perhaps his most remarkable ability was that he was able to recognize the relevance of new developments well ahead of most people. His desire was always to progress. A short summary of his fields of activity aptly demonstrates this.

(1) In 1954 Wilhelm Simon published his first paper on microtitration of organic compounds. The equipment he developed for this was in use for routine applications well into the 1980s.
(2) He began working with ion selective electrodes, the area for which he is probably best known, in the mid 1950s, when he carried out a number of projects with glass electrodes.
(3) In the early 1960s he developed equipment for the fully automatic determination of carbon, hydrogen and nitrogen; a commercial version of this apparatus was the leading product in this area for two decades.
(4) In the early 1960s he also developed a vapour pressure osmometer for the determination of the molar mass; again a successful commercial product resulted.
(5) In 1966 his group reported the combination of gas chromatography with mass spectrometry.
(6) He recognized the special effectiveness of the combination of diverse spectroscopical methods for the structural determination of organic compounds at a very early stage.
(7) The first use of antibiotics in ion selective electrodes was made by his group in 1966. In 1967 he was able to show that the molecular basis of ion pumps is a selective complexation of ions by these antibiotics. In 1968 he described corresponding selective transport of ions through artificial membranes.
(8) In 1969 he presented the potassium selective valinomycin electrode, a sensor that since then has been manufactured in hundreds of thousands of units. Today's clinical routine laboratories could not do without this sensor.
(9) He devoted much work to the structural determination of organic compounds, for example magneto-optical rotational dispersion and photo electron spectroscopy.
(10) Experimental and theoretical studies of ion selectivity in antibiotics were the basis of synthetic experiments for the recognition of selective analyte ions. Since 1972, well before the coining of such expressions as 'Wirt-Gast-Chemi' or 'supramolecular chemistry', over 1000 ionophores were synthesized by Wilhelm Simon's group and used in highly selective sensors for a number of ions. In 1975 he published details of the first enantiomeric selective sensor.
(11) In 1976 he published a paper on the development of ion selective micro electrodes (tip diameter $<1$ μm. These are used today to measure ion activity in living cells, as well as detectors in liquid chromatography and in capillary zone electrophoreses.
(12) One of Wilhelm Simon's goals was to develop chemical sensors so that they could be used in routine applications. His group worked on the improvement of membrane technology of sensors from the middle of the 1970s.
(13) The main activities in his last years were in the area of optical sensors. A new measurement principle, which was systematically used by his group, allowed the implementation of optical sensors for about a dozen analytes. In this, the ionophores which were developed earlier in his group were applied in combination with new lipophilic indicator compounds.

This impressive scientific performance was, of course, possible only with the aid of a substantial scientific staff. Professor Simon supervised 115 graduate students and 230 undergraduate students (Diplomanden). Seventy-two academics arrived from all over the world in order to be able to work with him. His enthusiasm was contagious; his optimism and endurance (which he learned during his youth when he was seriously ill) could not be impaired at any time. He never resigned after setbacks, but used to express his belief in a 'long-term justice'. He was also a talented speaker, in his classes and in his talks there was always a spark that jumped to the audience. His secret was a simple one: work. He prepared himself seriously and thoroughly for each class he taught and each discussion he led.

He was always available for everyone, and, in connection with personal problems, would encourage his staff to call him at all hours of the night. He never said no if someone approached him with a personal concern or was asking for something. He always provided a very liberal leadership to his staff. He never tried to talk someone out of a particular approach or did not permit something, even when he thought the planned experiments to be less than optimal.

His staff were his extended family. Several of the staff sports teams spontaneously chose the name 'Simonia'. In the 1970s this was a team for handball and successes were celebrated at his home. For each point won, one was allowed to pick a bottle from his wine cellar. Even later he participated in such celebrations after sports events wherever he could. If he did not have time, then they were entertained later. The last big 'family reunion' took place three years ago at the occasion of his 60th birthday. All former and current graduated students were invited. The guests said thank you with a 'poster session' in which each told about their career or about their memories in a poster.

When committed to a project, Wilhelm Simon worked on it without a break. Chemistry, and in particular analytical chemistry, was of central importance to him. He never chose the easiest way. At the Swiss Federal Institute of Technology (Eidgenössische Technische Hochschule Zürich, ETHZ), his career path ran as follows; 1956 thesis with Prelog and Heilbronner; 1960 habilitation thesis; 1965 assistant professor; 1967 extraordinary professor and 1970 ordinary professor for chemistry. In 1985 he got his professorship renamed to a professorship of analytical chemistry. Within the ETHZ he offered teaching classes in analytical chemistry, which normally are only made available with several professorships. He held many more classes and seminars than is normal. He was also able to find the financial support from third parties for teaching assistants. Until the very last he was battling for analytical chemistry, which particularly in recent times is endangered to loose terrain at various universities in Switzerland, in spite of the uncontested need for well-trained analytical chemists.

The world-wide acknowledgement of his merits has been demonstrated by a large number of honours, honourable memberships of professional societies and memberships in editorial boards or leading analytical chemistry journals (a total of 18).

His sudden health problems drastically interrupted an activity which was still far from fading away, he still had innumerable plans. With 20 graduate students, and many more staff, his laboratory was worldwide one of the most important centres in the area of chemical sensors. He leaves behind a big gap, in professional as well as human regard. Innumerable memories will live in all those that knew him.

Ernö Pretsch* and Jean Thomas Clerc†

* ETH-Zürich, Laboratorium für Organische Chemie, Universitätstrasse, 16 CH 8092 Zürich, Switzerland

† Universität Bern, Pharmazeutisches Institute, Baltzerstrasse 5, CH 3012 Bern, Switzerland

Translated for the *Journal of Automatic Chemistry* by Rolf W. Arndt, Mettler-Toledo AG, Im Langacher, CH 8606 Greifensee, Switzerland

